# Clinical differences between children with asthma and rhinitis in rural and urban areas

**DOI:** 10.25100/cm.v49i2.3015

**Published:** 2018-06-30

**Authors:** Jorge Sanchez, Andres Sánchez, Ricardo Cardona

**Affiliations:** 1 Group of Clinical and Experimental Allergy, IPS Universitaria Universidad de Antioquia. Medellin, Colombia; 2 Fundación para el Desarrollo de las Ciencias Médicas y Biológicas. Cartagena, Colombia; 3 Medicine Department, Corporacion Universitaria Rafael Nuñez, Cartagena, Colombia

**Keywords:** Asthma, children, diagnosis, treatment, rhinitis, rural, urban, asma, niños, diagnóstico, tratamiento, rinitis, rural, urbano

## Abstract

**Background:**

Epidemiological studies have shown that children who grow up on traditional farms are protected from allergic diseases. However, less is known about if the environment influences the pharmacotherapy in these patients.

**Objective:**

To compare the treatment of asthmatic and rhinitis children from urban and rural areas in Medellín, Colombia.

**Methods:**

During one year, we follow up a group of children (6 to 14 years) with diagnostic of asthma or rhinitis living for more than five years in urban or rural area. A questionnaire with socio-demographic characteristics, pharmacotherapy treatments, was obtained each three months. Atopy evaluation, spirometry and clinical test for asthma and rhinitis severity were done at the beginning and one year later.

**Results:**

Eighty six point four percent patients completed the follow up (rural n: 134, urban n: 248). Patients in rural location required less salbutamol (*p*: 0.01), visit to emergency department (*p* <0.01) and have a less number of patients with FEV1 <80% (*p*: 0.05). For clinical control rural children require less pharmacotherapy than urban children (*p*: 0.01) and more patients with rhinitis (18% vs 8% *p*: 0.03) and asthma (23% vs 12% *p*: 0.01) in the rural group could suspended pharmacotherapy. Atopy (*p*: <0.07) and poli-sensitization (*p*: <0.08) was a little higher in urban than rural area. We observe that poverty/unhygienic indicators were risk factors for higher levels of specific IgE among patients from urban area.

**Conclusion:**

Patients with respiratory allergies located in urban area require more pharmacotherapy and have less clinical response than rural children.

## Resumen

**Introducción:**

Los estudios epidemiológicos han demostrado que los niños que crecen en las granjas suelen tener menos frecuencia de enfermedades alérgicas. Sin embargo, se sabe menos si el tipo de ambiente (rural vs urbano) también puede influir en la respuesta clínica de a la farmacoterapia.

**Objetivo:**

Comparar un grupo de niños localizados en área rural y área urbana de Antioquia, Colombia, en cuanto al tratamiento farmacológico recibido para el asma y/o la rinitis.

**Métodos:**

Fueron incluidos niños con asma y/o rinitis que llevaran viviendo al menos 5 años en la misma zona rural o urbana con edades entre 6 a 14 años. A todos los pacientes se les realizó un seguimiento clínico cada 3 a 4 meses. La evaluación de la atopia, la espirometría y test para evaluar la gravedad del asma y la rinitis se realizaron al principio y al final del estudio.

**Resultados:**

De los pacientes candidatos, 382 (86.4%) completaron el seguimiento (rural n= 134 urbano n= 248). Los pacientes en área rural requirieron menos salbutamol (*p*: 0.01), visitas al departamento de emergencias (*p* <0.01) y tenían un menor número de pacientes con FEV1 <80% (*p* <0.05). Para el control clínico, los niños en zonas rurales requieren menos farmacoterapia que los niños en zona urbana (*p*: 0.01). Igualmente, para la rinitis (18% vs 8% *p*: 0.03) y el asma (23% vs 12% *p*= 0.01) un mayor número de los pacientes en zona rural pudieron suspender la farmacoterapia. La atopia (*p* <0.07) y la poli-sensibilización (*p* <0.08) fue mayor en las zonas urbanas que en las rurales. Se observó que los indicadores de pobreza y los servicios de aseo, eran factores de riesgo para mayores niveles de IgE entre los pacientes de área urbana.

**Conclusión:**

Los pacientes con asma o rinitis localizado en el área urbana tienen síntomas más severos y refractarios al tratamiento farmacológico, por lo que requieren más farmacoterapia que los niños rurales. Algunos factores ambientales intra y extra domiciliarios propios de la zona rural y urbana podrían influir en estos resultados.

## Introduction

Asthma and rhinitis are frequent respiratory diseases that affect all ages but with a marked predominance in children ^1^. The increasing prevalence of atopic disorders has been documented in many studies ^2^ ^,^ ^3^. Risk and protection factors have been studied, indicating that urbanized areas tend to have a higher incidence of disease compared to populations located in rural areas where prevalence is usually lower: In a population of Germany, the frequency of asthma and atopy was higher among patients living in the industrialized area than those who lived in less industrialized cities ^4^ ^,^ ^5^. These and other studies support that the environment could influence the development of allergies ^6^ ^,^ ^7^, but it has been less studied how urban environmental factors influence the severity of the clinical picture. Taking into account that people live in urban areas compared to those living in rural areas have a greater exposure to pro-inflammatory factors such as car smoke or chemical products from factories or processed foods ^8^ ^,^ ^9^, it could be assumed that the population living in cities have severe respiratory symptoms than the rural population. Although there are no studies that directly evaluate this hypothesis, some indirect results support it ^10^ ^,^ ^11^: high concentrations of O_3_, CO_2_, NOx, small carbon particles (PM <10) can cause hyperactivity in the respiratory tract inducing asthma or rhinitis, but also can alter the proteins of food and pollen grains ^12^ ^,^ ^13^, generating allergenic proteins with greater capacity to induce the production of IgE and the activation of T lymphocytes.

Achieving adequate asthma and rhinitis diagnosis and treatment in pediatric patients has become a key point in control of symptoms to prevent complication in adulthood ^14^: Treatment with regular inhaled corticosteroids, beta agonists, leukotrienes, and antihistamines are associated with improved control of symptoms. However, a greater severity of symptoms necessarily implies a greater dose of pharmacological treatment, a worse prognosis and an increased risk of adverse effects secondary to pharmacotherapy and lack of control of the disease. To our knowledge if the environmental conditions could change the treatment required for clinical control and the clinical consequences of this effect has not been studied. In this article, we evaluate if patients with asthma and / or rhinitis residents of urban and rural areas from Medellin, Colombia, have differences in the severity and treatment of asthma and rhinitis in terms of the dose required for the control. We also evaluate the evolution of patients over time focused on the response to treatment and the severity of respiratory diseases.

## Materials and Methods

### Population and geographic characteristics 

We created a community cohort for a prospective follow up and collection of epidemiological data and biological samples (RATA: Research about Tropical Trends in Asthma). The population of this study was collected in Antioquia - Colombia. The genetic background of rural and urban populations is the same and results from a racial admixture between Native Americans, Spaniards, and (albeit less frequently) Africans (<10.9%) ^15^ ^,^ ^16^. Antioquia is located in the Aburra Valley area (6º14’41’’ North, 75º340’29’’ West), 1,479 meters above sea level, with an average annual temperature of 22º C and RH: 66%. Children between 6 to 14 years old were randomly selected from December 2014 to January 2016 from two medical centers. Children living for more than 5 years in the same area (rural or urban) with a diagnosis of asthma or rhinitis according to GINA guideline ^17^ or ARIA guideline ^14^ without other respiratory or systemic were included. The study population was divided into “rural group” and “urban group” according to the census Bureau`s conditions ^18^ (https://www.census.gov/geo/reference/urban-rural.html). A questionnaire based in the ISAAC initiative to identify socio-demographic characteristics was done at the beginning of the study. A medical doctor expert in allergy diseases recruited all patients. The previous diagnosis, medicine treatment, and control of symptoms were investigated through questionnaire surveys written by the investigators based in the ISAAC questionnaire used in Colombia ^2^.

### Severity of symptoms and clinical control

To evaluate the severity of symptoms of asthma according to the perception of patients, we use the Asthma Control Test (ACT) previously validated in Colombia ^19^: this test have five (For people over 12 years of age) to seven (For people between 6 to 11 years of age) questions about common symptoms and patient control perception of asthma, rated on a scale of 0 to 25 points (>12 years) or 0 to 27 points (<11 years), are assessed according to the intensity. Depending of the age ACT considered “complete control” 25 or 27 points, “good control” 20-25 or 20-26 points, and “not control” <20 points. A spirometry was done at baseline and after 1 year of follow-up. Number of exacerbations, use of albuterol per week, visits to the emergency department were collected at each medical appointment (each 3 to 4 months).

To evaluate the severity of rhinitis we use the Allergic Rhinitis Symptom Questionnaire (ARSQ); the seven most common symptoms of rhinitis, rated on a scale of 0 to 4 points, are assessed according to the intensity (Absent to very severe). It is considered mild if the patient scores with 9 points or less, moderate from 10 to 19 points and severe from 20 to 28 points ^20^. 

### Pharmacotherapy evaluation

For asthma we register the pharmacotherapy require to clinical control in steps according to the GINA recommendations. Also, we assign for the therapy of each patient a score from 0 to 7 points according to the GINA steps (Supplement material, Table S1). For rhinitis, we register the pharmacotherapy requires to clinical control in agree to the ARIA steps recommendations. Also, we assign for the therapy of each patient a score from 0 to 4 points according to the ARIA steps (Table S2). During follow-up, patients without clinical control took an additional step in the management according to the medical criteria based on the GINA and ARIA guidelines. Patients with at least 3 months with clinical control had a reduction in the pharmacotherapy step according to the medical criteria. To avoid bias, physicians outside the study performed the medical evaluation of the patients.

### Follow-up

A spirometry was done at baseline and after 1 year of follow-up. Number of exacerbations, use of albuterol per week, visits to the emergency department, pharmacotherapy evaluation ACT and ARSQ were collected at each medical appointment (each 3 to 4 months).

### Ethical considerations

The Ethic Committee of the University of Antioquia from Medellín-Colombia approved this study. Because the participants were minors, the parents gave written informed consent. According to the request of the ethics committee, children also gave their assent, which was supervised by a child psychologist in children under 10 years of age.

### Analysis of data

Using double entry by three independent persons, all data were entered, categorized and analyzed using the Statistical Package for Social Sciences software (SPSS version 21, USA). Based on the data from a previous epidemiological survey and an analysis of children’s asthma in Colombia ^2^, the mean prevalence of childhood asthma in Medellin urban areas was 11% and for rhinitis 23%. There was not available data for rural area. With a level of confidence of 95%, power of 80%, and tolerance error of 0.5%, we estimated that 201 children from urban and 128 from rural area would be a sufficient number to estimate population statistics for the principal aim: compare the treatment of asthmatic and rhinitis children from urban and rural areas in Medellín, Colombia.

The Chi-square test was used to compare prevalence rates of disease severity and treatment between groups. The Mann-Whitney U was used to compare the control of symptoms between groups for abnormal distribution. Results are presented as 95% confidence intervals where appropriate. A *p* <0.05 was considered significant.

## Results

### Characteristics of the population

**Figure 1:**
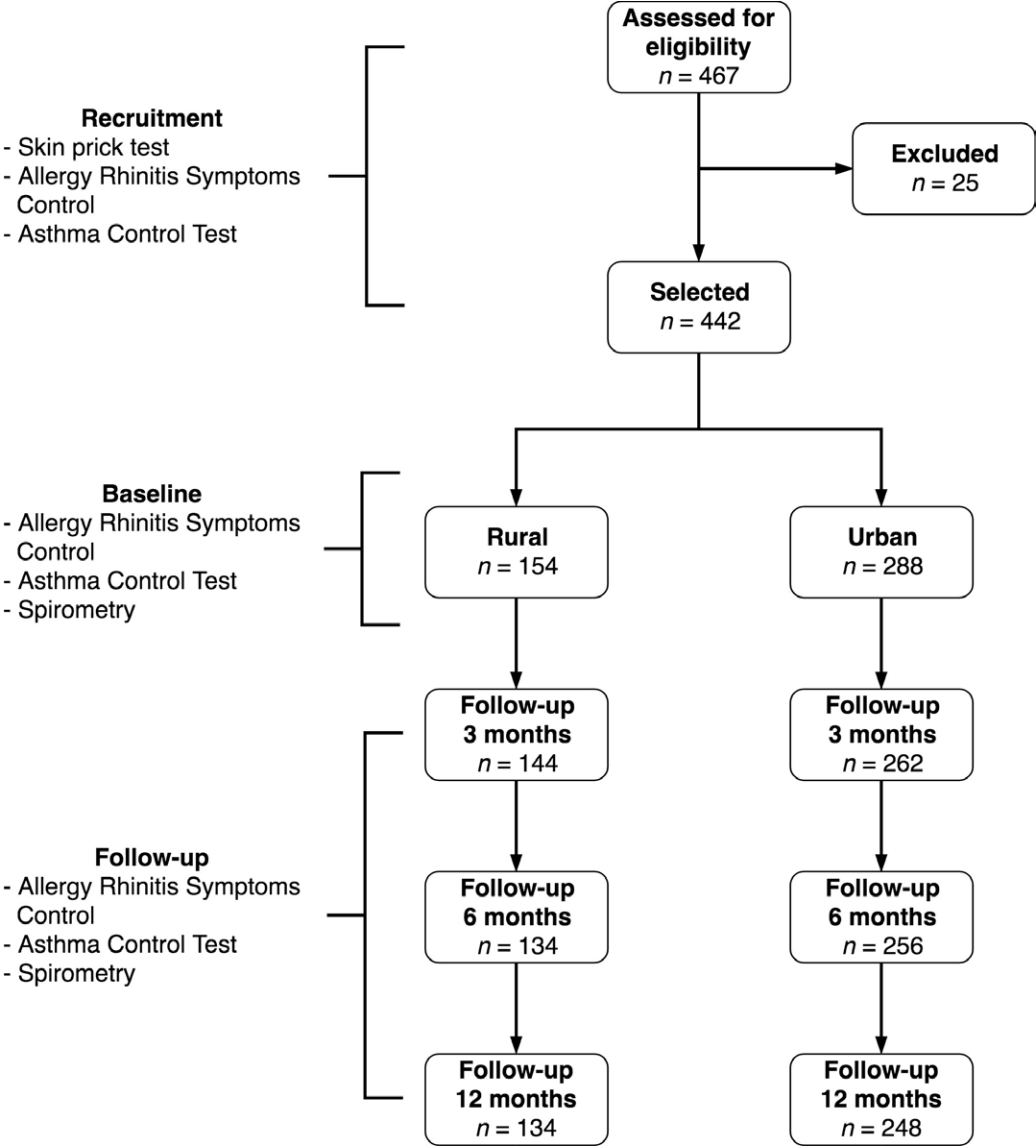
The flow chart represent the different moments of the study and the interventions during each step. ACT: Asthma Control Test. ARSQ: Allergy Rhinitis Symptoms Control.

From 467 patients that consult during the period of recruitment, 442 patients were selected and 382 (86.4%) finish the follow-up (Fig. 1 and Table 1): 134 from rural area and 248 from urban area. Reasons for drop-out were: moving out of study area (n= 11), loss of contact by inaccessible addresses (n= 23), lack of telephones (n= 16), social conflicts (n= 3), other reasons (n= 7). Families were lost in the interval of 0 to 6 months (Rural n= 20 and Urban n= 40). *Dermatophagoides* spp (83%), were the most important sensitizers followed by Dog (28%) and insects (24%). Sensitization to milk and egg were below 5%. Atopy (*p* <0.07) and poli-sensitization (*p* <0.08) was a little higher in urban than rural area, but it was not statistically significant.

**Table 1:** Socio-demographics characteristics.

Socio-demographics characteristics	Rural n (%)	Urban n (%)	*p*
Population n= 382	134 (35.1)	248 (64.9)	>0.10
Males n= 236 (61.7%)	80 (59.7)	156 (62.9)	>0.10
Age mean (range)	8 (6-14 SD: 4)	7 (6-14 SD: 5)	>0.10
Asthma	80 (59.7)	186 (75)	0.07
Rhinitis	112 (83.5)	224 (90.3)	0.09
Atopy	329 (86.1)	351 (91.8)	0.07
Natural Gas	120 (89.5)	234 (94.3)	>0.10
Electricity	124 (92.5)	248 (100.0)	0.08
Trash burning at home	24 (19.3)	10 (4)	**0.04**
Passive exposure to trash	28 (20.8)	20 (8)	**0.05**
Tap water	124 (92.5)	238 (95.9)	>0.10
Sewage	112 (83.5)	351 (91.8)	0.08
Houses of material	120 (89.5)	238 (95.9)	0.09
Covered floor	114 (85.0)	234 (94.3)	0.06
Socioeconomic strata (low 1 to 3)	130 (97.0)	218 (87.9)	**0.05**
ACT baseline	18 (2-27 SD: 8)	15 (2-27 SD: 14)	0.06
ARSQ baseline	12 (0-28 SD: 7)	16 (0-28 SD: 12)	**0.04**

ACT: Asthma Control Test.

ARSQ: Allergy Rhinitis Symptoms Control.

*p* <0.05 was statistically significant

The percentages are presented in parentheses

SD: Standard deviation

Most inhabitants living in urban and rural area were poor according to governmental indexes but most of them had assessed to basic services (Table 1). Trash burning at home was significantly most frequented in rural than in urban group. We did not observe that poverty/unhygienic indicators were risk factors for a higher severity of asthma or rhinitis, but it was associated with higher levels of specific IgE among patients from urban area and poli-sensitization (Urban n 43 sIgE Der p 131 kUA/mL + 62 Vs. Rural n 34 sIgE Der p 131 kUA/mL + 62; *p*: 0.02). There were not significant differences between patients who finish the study and those who drop out.

### ACT and ARSQ control

**Figure 2:**
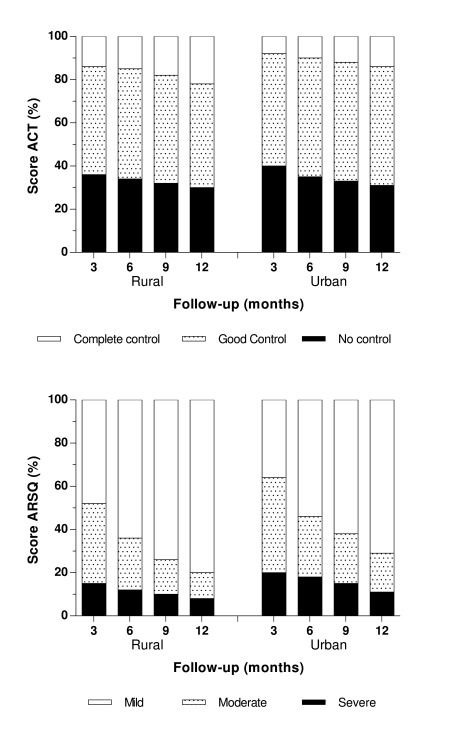
Percent of control according ACT and ARSQ are presented. ACT: Asthma Control Test. ARSQ: Allergy Rhinitis symptoms Control. P <0.05 was statistically significant.

At the baseline, patients in urban area had worse control of asthma and rhinitis according to ACT (*p*: 0.06) and ARSQ (*p*: 0.04) (Table 1). The clinical control according ACT for asthma was similar in both groups without significant differences, but there was a tendency of better clinical control in rural group (3 months *p*: 0.07, 6 months *p*: 0.08, 9 months *p*: 0.08, 12 months *p*: 0.7) (Fig. 2A). After 3 months, between 60 to 70% of patients present a complete or good control in both groups with a little increase over time but 30% of patients present not control even with an increase pharmacotherapy.

After 6 months and for the rest of the follow-up, there was a reduction in salbutamol requested and medical consultation in the emergency department (Table 2). This reduction was higher in the rural group. There was not significant difference between groups according mean FEV1 or FEV1/FVC ratio, but there were more patients in urban area with FEV1 lower than 80% in spirometry results (Table 2).

**Table 2:** Bronchial symptoms and spirometry.

	Rural n= 80 (59.7 %)	Urban n= 186 (75 %)	p
Days with Salbutamol per month: baseline/12months	4.8/2.4 (-50)	6.3/4.5 (-28.5)	0.01
Days in Emergency department: baseline/12months	1.4/0.4 (-71.4)	1.8/0.8 (-55.6)	<0.01
Baseline FEV1 (% predicted)	87+14	84+17	>0.10
Baseline FEV1<80% no. (%)	8 (10.0)	26 (13.9)	**0.05**
Baseline FEV1/FVC ratio	86.0 + 13.4	81.0 + 10.4	>0.10
1 year FEV1 (% predicted)	91.0 + 14.0	86.4+15.0	0.07
1 year FEV1<80% no. (%)	2.0 (2.5)	20.0 (10.7)	**0.05**
1 year FEV1/FVC ratio	88.0 + 13.4	85.0 + 10.4	>0.10

Spirometry was done at the beginning and after 12 months. Salbutamol and emergency department frequency was obtained in the first and the last medical visit.

The percentages are presented in parentheses

Similar results were presented according ARSQ for rhinitis, was similar in both groups with a tendency of better control in rural group but it was not statistically significant (3 Months *p*: 0.06, 6 months *p*: 0.08, 9 months *p*: 0.06, 12 months *p*: 0.8) (Fig. 2B). A large group of patients achieved adequate control for rhinitis than for asthma during follow-up and less than 12% in both rhinitis groups did not reach control at the end of the study.

### Pharmacotherapy response

**Figure 3:**
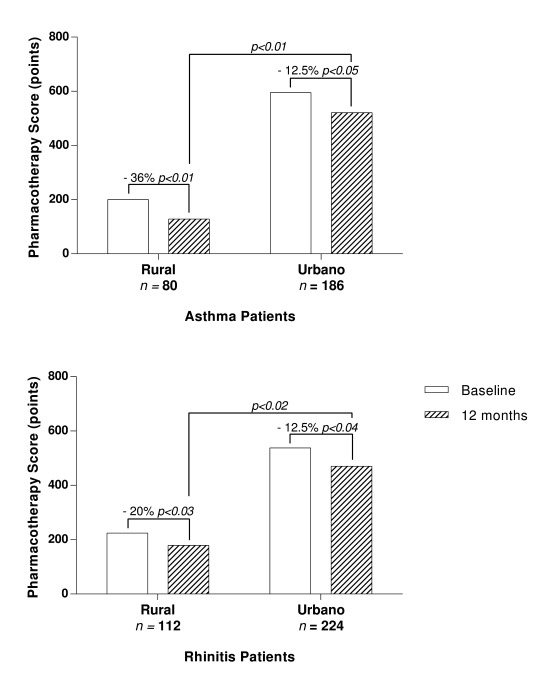
Comparison of pharmacotherapy score in asthma (Figure 3A) and rhinitis (Figure 3B). Changes in the pharmacotherapy during time (Baseline vs 12 months) and groups (Rural vs Urban) were compared. The percent numbers represent modifications in pharmacotherapy: negative values represent a reduction. *p* <0.05 was statistically

At the baseline, patients in urban area had higher severity than patients in rural area and requiring a higher dose of pharmacotherapy for asthma (Fig. 3): Rural group mean 2.5 (SD: 2) points vs. Urban group mean 3.2 (SD: 3) points respectively (*p*: 0.03). During the follow-up both groups have a reduction in pharmacotherapy being higher in the group of rural area: Rural area mean 1.6 (SD: 2) points vs. mean 2.8 (SD: 3) points respectively (*p*: 0.03). 

Also, after 12 months patients in rural area had a higher suspension of all drugs due to a complete clinical control for asthma (Fig. 3). 

For rhinitis after 6 months there was also a reduction maintained until the end of the study in pharmacotherapy been higher in rural group (baseline/12 months): Rural 2/1.6 (-20%) points and Urban 2.4/2.1 (-12.5%) points (Fig. 3). Suspension of rhinitis (18% vs 8% *p:* 0.03) and asthma (23% vs 12% *p*: 0.01) pharmacotherapy was also higher in rural group. 

The usage of alternative therapy, specific immunotherapy, antibiotics and Chinese medicine, were not significantly different between groups and had little change over time (*p* 0.18).

## Discussion 

To our knowledge this study is the first comparing the medical treatment for asthma and rhinitis between urban and rural areas in Latin America. In both rural and urban areas, there was a higher prevalence of asthma in males. This result is comparable to rural and Urban Chinese children population ^21^ and Spanish population ^22^.

A high exposure to irritants of the respiratory tract favors the chronicity of the inflammation and generates epithelial changes that worsen the picture and make its reversibility difficult ^23^. Clinically this translates into a greater severity of symptoms ^24^ ^,^ ^25^. We observed that the people located in the urban area needed a greater amount of medicines to achieve a good control of their symptoms, and they also have a greater number of emergency assistance and need for salbutamol than rural group, which indicates that urban population has more severe respiratory symptoms than patients living in the rural area. The reason for this tendency of lower asthma and rhinitis severity in rural areas and a higher tendency of atopy in urban area is unclear ^21^. In previous studies, exposure to agricultural farming and livestock farm conferred protection from asthma ^4^, may be because the greater exposure to various proteins in a natural way and less contact with various chemicals to which people in the city are usually exposed through air and processed food ^9^. Although these factors were not directly evaluated in this study, we assume that may influence our results: the level of contamination in Medellin is one of the worst in the country and since the geographic conditions of the Aburra Valley, it remains mainly concentrated in Urban area ^26^. In addition, the greater concentration of industries is located in the urban zone, whereas the agriculture and manufacture products is the main source of production in the rural area. According to the national poverty indexes, most of the population has low income; however in our study population more than 90% of the families included had a house made of material and adequate sewage conditions in both groups indicating that basic hygiene services are similar. Taking in consideration that genetic ancestry in both areas is the same ^15^ ^,^ ^16^, this suggest that environmental factors and maybe cultural factors are more important to explain these differences like air pollution as we previously suggest. Patients in rural area had a higher exposure to trash burning, although we did not find significant differences between the exposed and non-exposed groups (*p* >0.1).

According to GINA and ARIA guideline, treatment with a regular daily dose of steroids is highly effective in reducing symptoms and reducing the risk of asthma exacerbation. However, similar to Chinese studies ^21^, according to ACT only 30% of asthmatic children in rural and urban area had complete control and 30% had no control even with additional therapies. One possible explanation is that ACT had not enough sensitivity to differentiate the level of control; this is supported because according to Analog Visual Scale most patients reported good clinical control. Nevertheless, ACT has objective questions that have been previously validated ^19^. This suggests that current therapies are not enough effective to achieve complete asthma control in a significant number of patients ^27^. Other reasons like poor adherence, low availability of drugs or pre-study management of asthma could also contribute to the low response. Even with these considerations, patients in the rural group required lower pharmacotherapy than urban area for a good control in asthma and rhinitis. Also a significant number of patients in the rural group could suspend drugs and required a lower use of salbutamol, assistance to the emergency department and had a better spirometry results before and after pharmacotherapy. 

Rhinitis is the most common respiratory disease ^28^. Some studies have observed that their impact on quality of life may be higher than that caused by asthma ^29^. We observed that clinical control for rhinitis was higher than asthma but the number of patients with a complete suspension of nasal treatment was lower. Considering that a significant number of patients achieve a remission of bronchial symptoms before puberty, our results would indicate that this remission does not apply for nasal symptoms since in patients with asthma and bronchial remission was not necessary accompanied by a remission of rhinitis. Even with a lower rate of remission, similar to asthma, patients in rural area had a better control of rhinitis and tolerated a higher reduction of pharmacotherapy suggesting, again, some urban environmental factors. 

Our results have several implications. In addition to the clinical aspects that the patient suffers, the lack of control has for them and their family a great social and economic impact: children with uncontrolled respiratory symptoms, have a lower school performance and and may have personality disorders ^30^. A lack of control of the symptoms results in a polypharmacy of the patient, with a greater economic cost for the health system and the family, together with the greater risk of adverse events ^30^ ^,^ ^31^. Therefore, the study of the factors that lead to greater severity of asthma and rhinitis are necessary, especially the identification of those factors that may be modifiable and reduce the social and economic impact of the family.

Our study has some limitations. The sociocultural and environmental conditions of each region limit the extrapolation of our results to other populations ^6^. However, the results of the ISAAC study indicate that many of the risk factors for asthma are present throughout the world ^1^ ^,^ ^32^. It is therefore necessary to do similar studies in other populations to evaluate the reproducibility of our results, and identify the main associated factors. Because our study was conducted in children, we do not evaluate whether the greater severity of asthma and rhinitis in the urban area extends to adulthood. Our hypothesis is that yes, since the chronic inflammation at the level of the respiratory tract during infancy could generate changes that would not revert in the adulthood ^33^.

## Conclusion

Compared with rural area, urban location has important consequences in the severity and control of asthma and rhinitis which are not full reversed despite the use of increased pharmacotherapy. The identification of the environmental risk factors associated with these results could help to improve the control of asthma in urban and rural areas. 

## Supplementary material

**Table S1:** Pharmacotherapy points according GINA steps. 2 times for week 1 point

GINA step	First Choice	Second Choice	Score
Step 1	No need continuous control therapy		0 point
	low dose ICS <2 times for week.- SABA >2 times for week	1 point
Step 2	Low dose ICS	LTRA. Low dose theophyline	2 points
Step 3	Low dose ICS/LABA	Medium dose of ICS dose	3 points
	High dose of ICS dose. - Low dose ICS/LABA +LTRA (or +theophylline)	4 points
Step 4	Medium dose of ICS		4 points
Step 5	Tiotropium*. - Omalizumab - Mepoluzumab		6 points
	Add low dose OCS	7 points

LTRA: Leukotriene receptor antagonists. SABA: Short-acting beta2-agonist; ICS: inhaled corticosteroids; OCS: Oral corticosteroids; LABA: long-acting beta2-agonist; med: medium dose; OCS: oral corticosteroids

Low, medium, and high dose of the different ICS was defined according to GINA guideline. Tiotropium was only for children over 12 years.

## Supplementary material

**Table S2:** Pharmacotherapy points according ARIA steps.

ARIA step	First Choice	Second Choice	Score
Mild intermittent	Oral anti-H1 intermittent (<2 days per week)	Intra-nasal or oral decongestant (<10 days per month)	0 point
Oral anti-H1 intermittent (>3 days per week)		1 point
Moderate/severe Intermittent	Intra-nasal steroid	Local chromone	2 points
Mild persistent	Intra-nasal steroid and antiH1	Intra-nasal-steroid and AntiL	3 points
Moderate/severe Persistent	Intra-nasal steroid + antiH1 + antiL	4 points

AntiH1: Antihistamine
