# Supplementary material for: Clinical differences between children with asthma and rhinitis in rural and urban areas
Source: Colomb Med (Cali). 2018 Jun 30;49(2):169–74. doi: 10.25100/cm.v49i2.3015 (PMC6084920; doi:10.25100/cm.v49i2.3015)
Supplement: Related texts

# Research article: Diferencias clínicas entre niños con asma y rinitis de áreas rurales y urbanas

## Introducción

El asma y la rinitis son enfermedades respiratorias frecuentes que afectan a todas las edades, pero son predominantes en los niños ^1^. La creciente prevalencia de trastornos atópicos se ha documentado en muchos estudios ^2^^,^^3^. Diferentes autores han estudiado los factores de riesgo y protección, encontrando que las áreas urbanizadas tienden a tener una mayor incidencia de enfermedades alérgicas en comparación con las poblaciones ubicadas en zonas rurales donde la prevalencia suele ser menor: en una población de Alemania, la frecuencia de asma y atopia en el área industrializada era mayor que aquellos que vivían en ciudades menos industrializadas ^4^^,^^5^. Estos y otros estudios respaldan que el ambiente podría influir en el desarrollo de alergias ^6^^,^^7^, pero se ha estudiado poco cómo los factores ambientales urbanos influyen en la gravedad del cuadro clínico. Teniendo en cuenta que las personas que viven en áreas urbanas comparadas con aquellas que viven en áreas rurales tienen una mayor exposición a factores proinflamatorios como el humo de automóviles o productos químicos de fábricas o alimentos procesados ^8^^,^^9^, se puede suponer que la población que vive en las ciudades tiene síntomas respiratorios más graves que la población rural. Aunque no hay estudios que evalúen directamente esta hipótesis, algunos resultados indirectos lo respaldan ^10^^,^^11^: altas concentraciones de O_3_, CO_2_, NO_x_ y pequeñas partículas de carbono (PM <10) pueden causar hiperactividad en el tracto respiratorio induciendo asma o rinitis, pero también puede alterar las estructuras de las proteínas de los alimentos y los granos de polen ^12^^,^^13^, generando proteínas alergénicas con mayor capacidad para inducir la producción de IgE y la activación de los linfocitos T.

Hoy en día, lograr un diagnóstico y tratamiento adecuado del asma y la rinitis en pacientes pediátricos se ha convertido en un punto clave en el control de los síntomas para prevenir complicaciones en la edad adulta ^14^: el tratamiento con corticosteroides inhalados regulares, beta-agonistas, leucotrienos y antihistamínicos está asociado con un mejor control de los síntomas. Sin embargo, una mayor gravedad de la enermedad implica necesariamente una mayor dosis en el tratamiento farmacológico, lo que lleva a un peor pronóstico y un mayor riesgo de efectos secundarios adversos a la farmacoterapia y la falta de control de la enfermedad. Sin embargo, aún no se conoce con claridad si las condiciones ambientales pueden cambiar el tratamiento requerido para el control clínico y las consecuencias clínicas del paciente. En este artículo, evaluamos si los pacientes con asma y / o rinitis residentes en áreas urbanas y rurales de Medellín, Colombia, tienen diferencias en la gravedad y el tratamiento del asma y la rinitis en términos de la dosis requerida para el control. También evaluamos la evolución de los pacientes a lo largo del tiempo centrada en la respuesta al tratamiento y la gravedad de las enfermedades respiratorias.

## Materiales y Métodos

### Población y características geográficas

Creamos una cohorte comunitaria para hacer seguimiento prospectivo y una recopilación de datos epidemiológicos y muestras biológicas (RATTA: investigación sobre tendencias tropicales en el asma). La población de este estudio fue recolectada en Antioquia - Colombia. El trasfondo genético de las poblaciones rurales y urbanas es el mismo y es el resultado de una mezcla racial entre nativos americanos, españoles y (aunque con menos frecuencia) africanos (<10.9%) ^15^^,^^16^. Antioquia está ubicada en el área del Valle de Aburrá (6º14'41'' Norte, 75º340'29'' Oeste), a 1,479 metros sobre el nivel del mar, con una temperatura media anual de 22º C y RH: 66%. Los niños de entre 6 y 14 años de edad fueron seleccionados aleatoriamente entre diciembre de 2014 y enero de 2016 de dos centros médicos. Se incluyeron niños que residen hace más de cinco años en la misma área (rural o urbana) con un diagnóstico de asma o rinitis de acuerdo con la guía GINA ^17^ o la guía ARIA ^14^, sin otro tipo de problemas respiratorios o sistémicos. La población de estudio se dividió en "grupo rural" y "grupo urbano" de acuerdo con las condiciones del sistema de censo Buró ^18^ (https://www.census.gov/geo/reference/urban-rural.html). Al comienzo del estudio se realizó un cuestionario basado en la iniciativa ISAAC para identificar las características sociodemográficas. El reclutamiento de los pacientes se realizó por médicos especialistas en alergología. El diagnóstico anterior, el tratamiento médico y el control de los síntomas se investigaron a través de cuestionarios basados en el cuestionario ISAAC utilizado en Colombia ^2^.

### Gravedad de los síntomas y control clínico

Para evaluar la gravedad de los síntomas del asma según la percepción de los pacientes, utilizamos la prueba de control del asma (ACT) previamente validada en Colombia ^19^: esta prueba tiene entre cinco (para personas mayores de 12 años) hasta siete preguntas (para personas entre 6 y 11 años de edad) sobre los síntomas comunes y la percepción del control del asma por parte del paciente, calificadas en una escala de 0 a 25 puntos (>12 años) o de 0 a 27 puntos (<11 años), basándose en la intensidad de los síntomas. Dependiendo de la edad, el ACT fue considerado como "control total" con 25 o 27 puntos, "buen control" con 20-25 o 20-26 puntos, y "no control" cuando era <20 puntos. Se realizó una espirometría al inicio del estudio y después de un año de seguimiento. También se recolectaron en cada cita médica (cada 3 a 4 meses) el número de exacerbaciones, frecuencia de uso de salbutamol por semana.

Para evaluar la gravedad de la rinitis, usamos el Cuestionario de Síntomas Alérgicos de la Rinitis (ARSQ); en la escala se evalúa los síntomas más comunes de la rinitis, clasificados en una escala de 0 a 4 puntos, se evaluaron según la intensidad (de ausente a muy grave). Se consideró leve si el paciente puntúa con 9 puntos o menos, moderado de 10 a 19 puntos y severo de 20 a 28 puntos ^20^.

### Evaluación de farmacoterapia

Para el asma, registramos la farmacoterapia requerida para el control clínico de acuerdo con las recomendaciones del GINA. Además, asignamos para la terapia de cada paciente una puntuación de 0 a 7 puntos según los pasos de GINA (material suplementario, Tabla S1). Para la rinitis, registramos la farmacoterapia de acuerdo a los pasos de control del ARIA (Tabla S2). Durante el seguimiento, los pacientes sin control clínico continuaron el aumento escalonado de la farmacoterapia de acuerdo a la recomendaciones de las guías GINA y ARIA. Los pacientes con al menos 3 meses con control clínico tuvieron una reducción en el paso de farmacoterapia de acuerdo con los criterios médicos. Para evitar el sesgo, médicos externos al estudio realizaron la evaluación médica de los pacientes.

### Seguimiento

Se realizó una espirometría al inicio del estudio y después de 1 año de seguimiento. se obtuvieron en cada cita médica el número de exacerbaciones, uso de salbutamol (albuterol) por semana, visitas al servicio de urgencias y la evaluación de farmacoterapia ACT y ARSQ (cada 3 a 4 meses).

### Consideraciones éticas

El estudio fue aprobado por el Comité de Ética de la Universidad de Antioquia de Medellín, Colombia. Debido a que los participantes eran menores de edad, los padres dieron su consentimiento informado por escrito para su inclusión en el estudio. De acuerdo con la solicitud del comité de ética, los pacientes también dieron su consentimiento el cuál fue supervisado por un psicólogo infantil en niños menores de 10 años.

### Análisis de los datos

Se realizó un doble chequeo de los datos por tres personas de forma independientes, todos los datos se ingresaron, categorizaron y analizaron utilizando el paquete “*Statistical Package for Social Sciences*” (SPSS versión 21, EE.UU.). Cbase en los datos de una encuesta epidemiológica previa y un análisis del asma infantil en Colombia ^2^, la prevalencia promedio de asma infantil en las áreas urbanas de Medellín fue del 11% y para la rinitis del 23%. No hubo datos disponibles para el área rural. Con un nivel de confianza del 95%, una potencia del 80% y un error de tolerancia del 0.5%, estimamos que 201 niños de zonas urbanas y 128 del área rural serían un número suficiente para estimar las estadísticas de población con el objetivo principal: comparar el tratamiento de niños con asma y rinitis de áreas urbanas y rurales en Medellín, Colombia.

La prueba de Chi-cuadrado se utilizó para comparar las tasas de prevalencia de la gravedad de la enfermedad y el tratamiento entre los grupos. Se usó la U de Mann-Whitney para comparar el control de los síntomas entre los grupos para una distribución anormal. Los resultados se presentan como intervalos de confianza del 95% cuando corresponde. Una *p* <0.05 se consideró estadísticamente significativa.

## Resultados

### Características de la población

De los 467 pacientes que consultaron durante el período de reclutamiento, se seleccionaron 442 pacientes y de estos 382 (86.4%) finalizaron el seguimiento (Fig. 1 y Tabla 1): 134 del área rural y 248 del área urbana. Las razones por la cual abandonaron el estudio fueron: mudarse fuera del área de estudio (n= 11), pérdida de contacto por direcciones inaccesibles (n= 23), falta de teléfonos (n= 16), conflictos sociales (n= 3), otras razones (n= 7). Estas familias se perdieron en un intervalo de 0 a 6 meses (Rural n= 20 y Urbano n= 40). *Dermatophagoides* spp (83%), fueron la fuente de sensibilización más importante, seguido por el perro (28%) e insectos (24%). La sensibilización a la leche y al huevo fue inferior al 5%. La atopia (*p* <0.07) y la polisensibilización (*p* <0.08) fueron un poco más altas en áreas urbanas que rurales, pero no fueron estadísticamente significativas.

**Figura 1:** El diagrama de flujo representa los diferentes momentos del estudio y las intervenciones durante cada paso. ACT: prueba de control del asma. ARSQ: Control de los síntomas de la rinitis alérgica.

**Tabla 1:** Características Socio-demográficas.

Características Socio-demográficas	Rural n (%)	Urbano n (%)	*p*
Población n= 382	134 (35.1)	248 (64.9)	>0.1
Hombres n= 236, 61.7%	80 (59.7)	156 (62.9)	>0.1
Edad media (rango)	8 (6-14 SD 4)	7 (6-14 SD 5)	>0.1
Asma	80 (59.7)	186 (75)	0.07
Rinitis	112 (83.5)	224 (90.3)	0.09
Atopia	329 (86.1)	351 (91.8)	0.07
Gas natural	120 (89.5)	234 (94.3)	>0.1
Electricidad	124 (92.5)	248 (100)	0.08
Basura quemada en casa	24 (19.3)	10 (4)	**0.04**
Exposición pasiva a basuras	28 (20.8)	20 (8)	**0.05**
Agua de grifo	124 (92.5)	238 (95.9)	>0.1
Aguas residuales	112 (83.5)	351 (91.8)	0.08
Casas de material	120 (89.5)	238 (95.9)	0.09
Piso cubierto	114 (85.0)	234 (94.3)	0.06
Estrato Socioeconómico (1 a 3)	130 (97.0)	218 (87.9)	**0.05**
ACT basal	18 (2-27 DE: 8)	15 (2-27 DE: 14)	0.06
ARSQ basal	12 (0-28 DE: 7)	16 (0-28 DE: 12)	**0.04**

ACT: prueba de control del asma.

ARSQ: Control de los síntomas de la rinitis alérgica.

Una *p* <0.05 fue estadísticamente significativo

Los porcentajes se presentan entre paréntesis

DE: Desviación estándar

La mayoría de los habitantes que vivían en áreas urbanas y rurales eran pobres según los índices gubernamentales, pero la mayoría de ellos tenían accesos a los servicios básicos (Tabla 1). La basura quemada en casa era significativamente más frecuente en el grupo rural que en el urbano. No observamos que los indicadores de pobreza / antihigiénicos fueran factores de riesgo para una mayor gravedad de asma o rinitis, pero se asoció con mayores niveles de IgE específica entre pacientes del área urbana y polisensibilización (datos no mostrados). No hubo diferencias significativas entre los pacientes que terminaron el estudio frente a los que abandonaron.

### Control ACT y ARSQ

Al inicio, los pacientes en el área urbana presentaron un menor control del asma y la rinitis de acuerdo con el ACT (*p*: 0.06) y ARSQ (*p*: 0.04) (Tabla 1). El control clínico según ACT para el asma fue similar en ambos grupos, sin presentar diferencias significativas, pero hubo una tendencia en tener un mejor control clínico en los pacientes del grupo rural (3 meses *p*: 0.07, 6 meses *p*: 0.08, 9 meses *p*: 0.08, 12 meses *p*: 0.07) (Fig. 2A). Después de 3 meses, entre el 60 y el 70% de los pacientes presentaron un buen control o un control completo en ambos grupos con mejoría a través del tiempo, sin embargo, el 30% de los pacientes no presentaron un control y requirieron un aumento del uso de la farmacoterapia.

**Figura 2:** Porcentaje de control de acuerdo a ACT y ARSQ. ACT: prueba de control del asma. ARSQ: Control de los síntomas de la rinitis alérgica. La *p* <0.05 fue estadísticamente significativa.

Después de 6 meses y durante el resto del seguimiento, hubo una reducción en el requerimiento de salbutamol y en la asistencia al departamento de emergencias (Tabla 2). Esta disminución fue más acentuada en el grupo rural. Además, no hubo diferencias significativas entre los grupos en los resultados de espirometría según la relación FEV1 media o FEV1 / FVC, pero hubo más pacientes en el área urbana con FEV1 inferior al 80% (Tabla 2).

**Tabla 2:** Exacerbaciones y función pulmonar.

	Rural n= 80 (59.7%)	Urbano n= 186 (75%)	p
Días con salbutamol por mes: Inicial/12 meses	4.8/2.4 (-50)	6.3/4.5 (-28.5)	0.01
Días en departamento de urgencias: Inicial/12 meses	1.4/0.4 (-71.4)	1.8/0.8 (-55.6)	<0.01
Inicial FEV1 (% predictivo)	87+14	84+17	>0.1
Inicial FEV1 <80% no. (%)	8 (10.0)	26 (13.9)	**0.05**
Inicial FEV1/FVC radio	86 + 13.4	81 + 10.4	>0.1
1 año FEV1 (% predictivo)	91 + 14	86,4+15	0.07
1 año FEV1<80% no. (%)	2 (2.5)	20 (10.7)	**0.05**
1 año FEV1/FVC radio	88 + 13.4	85 + 10.4	>0.1

La espirometría se realizó al comienzo y después de 12 meses. El uso de salbutamol y la frecuencia de asistencia al servicio a urgencias se obtuvieron en la primera y la última consulta médica.

Los porcentajes se presentan entre paréntesis

Con respecto a la rinitis, se presentaron resultados similares según el ARSQ en ambos grupos, sin embargo, en el grupo rural se observó una ligera tendencia a un mejor control, pero no fue estadísticamente significativa (3 meses *p*: 0.06, 6 meses *p*: 0.08, 9 meses *p*: 0.06, 12 meses *p*: 0.08) (Fig. 2B). Un gran número de pacientes durante el seguimiento tuvieron un control más adecuado para la rinitis que para el asma y menos del 12% en ambos grupos de pacientes con rinitis no alcanzaron el control al final del estudio.

### Respuesta farmacoterapia

Al inicio, los pacientes en el área urbana tenían mayor gravedad que los pacientes en el área rural y requerían una dosis más alta de farmacoterapia para el asma (Fig. 3): Media del grupo de área rural 2.5 puntos (DS: 2) vs. media del grupo de área urbana 3.2 puntos (DS: 3) (*p*: 0.03). Durante el seguimiento, ambos grupos presentaron una reducción en la farmacoterapia siendo más alta en el grupo del área rural: Media del grupo de área rural 1.6 puntos (DS: 2) vs. media del grupo de área urbana 2.8 puntos(DS: 3), respectivamente (*p*: 0.03).

**Figura 3:** Comparación de la puntuación de farmacoterapia en el asma (A) y la rinitis (B). Se compararon los cambios en la farmacoterapia a lo largo del tiempo (inicial versus 12 meses) y grupos (rural versus urbano). Los porcentajes representan modificaciones en la farmacoterapia: los valores negativos representan una reducción. La *p* <0.05 fue estadísticamente significativa.

Además, después de 12 meses, los pacientes en el área rural tenían una mayor suspensión de todos los medicamentos debido a un control clínico completo del asma (Fig. 3).

Para el caso de los pacientes con rinitis, después de 6 meses también se observó una reducción en el uso de farmacoterapia hasta el final del estudio, siendo mayor la reducción en el grupo rural (0 meses / 12 meses): Rural 2 / 1.6 (-20%) puntos y urbano 2.4 / 2.1 (-12.5%) puntos (Fig. 3). La suspensión de la farmacoterapia para la rinitis (18% frente a 8% *p*: 0.03) y el asma (23% frente a 12% *p*: 0.01) también fue mayor en el grupo rural.

El uso de terapia alternativa, inmunoterapia específica, antibióticos y medicina china, no fue significativamente diferente entre los grupos estudiados y tuvo pocos cambios a lo largo del tiempo (*p*: 0.18).

## Discusión

A nuestro conocimiento, este estudio es el primero que compara el tratamiento médico para el asma y la rinitis entre áreas urbanas y rurales en América Latina. En las áreas rurales y urbanas, hubo una mayor prevalencia de asma en el sexo masculino. Este resultado es comparable a lo observado en la población rural y urbana de niños chinos y a la población española ^21^^,^^22^.

Una alta exposición a los irritantes del tracto respiratorio favorece la cronicidad de la inflamación y genera cambios epiteliales que empeoran la enfermedad y dificultan su reversibilidad ^23^. Clínicamente esto se traduce en una mayor gravedad de los síntomas ^24^^,^^25^. Encontramos que las personas ubicadas en el área urbana necesitan una mayor cantidad de medicamentos para lograr un buen control de sus síntomas, y también presentan un mayor número de asistencia de emergencia y necesidad de salbutamol que el grupo rural, lo que indica que la población urbana tiene más síntomas respiratorios graves que los pacientes que viven en el área rural. La razón de esta tendencia a menor gravedad de la rinitis y el asma en las zonas rurales y una mayor tendencia de la atopia en el área urbana no está definida ^21^. Estudios previos han observado, que la cercanía a las granjas de agricultura y ganadería confiere una protección contra el asma ^4^, lo cual puede deberse a una mayor exposición contra diversas proteínas de forma natural y una disminución en el contacto con diversos productos químicos a los que las personas en la ciudad suelen estar expuestas por aire y alimentos procesados que favorecen la gravedad de estas enfermedades respiratorias ^9^. Aunque estos factores no se evaluaron directamente en este estudio, suponemos que pueden influir en nuestros resultados: el nivel de contaminación en Medellín es uno de los peores de la región y debido a las condiciones geográficas del Valle de Aburrá, se mantiene principalmente concentrado en el área urbana ^26^. Además, la mayor concentración de industrias se ubica en la zona urbana, mientras que la agricultura y los productos de manufactura son la principal fuente de producción en el área rural. Según los índices nacionales de pobreza, la mayoría de la población tiene bajos ingresos, sin embargo, en nuestro estudio más del 90% de las familias incluidas tenían una casa hecha de material y condiciones adecuadas de drenaje en ambos grupos, lo que indica que los servicios básicos de higiene son similares. Teniendo en cuenta que la ascendencia genética en ambas áreas es la misma ^15^^,^^16^, esto sugiere que los factores ambientales y tal vez los factores culturales son más importantes para explicar estas diferencias, como la contaminación del aire, que hemos sugerido anteriormente. Los pacientes en el área rural tuvieron una mayor exposición a la quema de basura, aunque no encontramos diferencias significativas entre los grupos expuestos y no expuestos (*p* >0.1).

Según las guías GINA y ARIA, el tratamiento con una dosis diaria regular de esteroides es altamente efectivo para reducir los síntomas y reducir el riesgo de exacerbación del asma. Sin embargo, de manera similar a los estudios en chinos ^21^, según el ACT, solo el 30% de los niños asmáticos en áreas rurales y urbanas tenían control completo y 30% no tenían un control incluso con terapias adicionales. Una posible explicación es que el ACT no tiene suficiente sensibilidad para diferenciar el nivel de control y esto estaría apoyado en que, de acuerdo a la escala visual análoga, la mayoría de los pacientes informaron un buen control clínico. Sin embargo, ACT tiene preguntas objetivas que han sido previamente validadas ^19^, lo que sugiere que las terapias actuales no son lo suficientemente efectivas para lograr el control completo del asma en un número significativo de pacientes ^27^. Otras razones como la falta de adherencia, la baja disponibilidad de medicamentos o el tipo manejo previo al estudio del asma también podrían contribuir a la baja respuesta. Incluso con estas consideraciones, los pacientes del área rural requirieron un menor uso de la farmacoterapia que los pacientes del área urbana para un buen control en el asma y la rinitis. Además, un número significativo de pacientes del grupo rural suspendieron los medicamentos y requirieron un menor uso de salbutamol, así como una disminución en la asistencia al departamento de emergencia y tuvieron mejores resultados de espirometría antes y después de la farmacoterapia.

La rinitis es la enfermedad respiratoria más común ^28^. Algunos estudios han observado que su impacto en la calidad de vida puede ser mayor que el causado por el asma ^29^. En nuestro estudio observamos que el control clínico de la rinitis era más alto que el del asma, pero el número de pacientes con una suspensión completa del tratamiento nasal fue menor. Teniendo en cuenta que un número significativo de pacientes tienen una remisión de síntomas bronquiales antes de la pubertad, nuestros resultados indican que esta remisión no se aplica a los síntomas nasales ya que en pacientes con asma con remisión bronquial no estaban necesariamente acompañados de una remisión de la rinitis. Incluso con una tasa de remisión más baja, similar al asma, los pacientes del área rural tenían un mejor control de la rinitis y toleraron una mayor reducción de la farmacoterapia lo que sugiere, una vez más, la influencia de algunos factores ambientales urbanos.

Nuestros resultados tienen varias implicaciones. Además de los aspectos clínicos que sufre el paciente, la falta de control tiene para ellos y su familia un gran impacto social y económico: los niños con síntomas respiratorios no controlados, tienen un rendimiento escolar más bajo y pueden tener trastornos de la personalidad ^30^. La falta de control de los síntomas resulta en una polifarmacia del paciente, con un mayor costo económico para el sistema de salud y la familia, junto con un mayor riesgo de eventos adversos ^30^^,^^31^. Por lo tanto, es necesario el estudio de los factores que conducen a una mayor gravedad del asma y la rinitis, especialmente la identificación de aquellos factores que pueden ser modificables y reducir el impacto social y económico de la familia.

Nuestro estudio tiene algunas limitaciones. Las condiciones socioculturales y ambientales de cada región limitan la extrapolación de nuestros resultados a otras poblaciones ^6^. Sin embargo, los resultados del estudio ISAAC indican que muchos de los factores de riesgo para el asma están presentes en todo el mundo ^1^^,^^32^. Por lo tanto, es necesario realizar estudios similares en otras poblaciones para evaluar la reproducibilidad de nuestros resultados e identificar los principales factores asociados. Debido a que nuestro estudio se realizó en niños, no evaluamos si la mayor gravedad del asma y la rinitis en el área urbana se extiende hasta la edad adulta. Nuestra hipótesis es que sí, ya que la inflamación crónica a nivel del tracto respiratorio durante la infancia podría generar cambios que no se revertirían en la edad adulta ^33^.

## Conclusión

En comparación con el área rural, la ubicación urbana tiene importantes consecuencias en la gravedad y el control del asma y la rinitis, que no se controlan por completo a pesar del uso de la farmacoterapia. La identificación de los factores de riesgo ambientales asociados con estos resultados podría ayudar a mejorar el control del asma en áreas urbanas y rurales.

## MATERIAL SUPLEMENTARIO

**Tabla S1:** Puntos de farmacoterapia de acuerdo a los pasos GINA. 2 veces por semanda 1 punto

Pasos GINA	Primera opción	Segunda opción	Puntaje
Paso 1	No necesita una terapia de control continua		0 puntos
	Baja dosis ICS <2 veces por semana - SABA >2 veces por semana	1 punto
Paso 2	Dosis baja de ICS	LTRA. Dosis baja de theophyline	2 puntos
Paso 3	Baja dosis de ICS/LABA	Dosis media de ICS	3 puntos
	Dosis alta de ICS. - Dosis baja de ICS/LABA +LTRA (o +theophylline)	4 puntos
Paso 4	Dosis media de ICS		4 puntos
Paso 5	Tiotropium*. - Omalizumab - Mepoluzumab		6 puntos
	Adicione una disis baja de OCS	7 puntos

LTRA: Receptor antagonista de Leukotriene. SABA: Short-acting beta2-agonist; ICS: Corticosteroides inhalados; OCS: Corticosteroides orales; LABA: long-acting beta2-agonist; OCS: Oral corticosteroids

Las dosis baja, media y alta ICS fueron definidas de acuerdo a la guia GINA. Tiotropium se dio solo para niños >12 años.

## MATERIAL SUPLEMENTARIO

**Tabla S2:** Puntos de farmacoterapia de acuerdo a los pasos ARIA.

Pasos ARIA	Primera opción	Segunda opción	Puntaje
Leve intermitente	Anti-H1 intermitente oral (<2 veces por semana)	Decongestionante intra-nasal u oral (<10 dias por mes)	0 puntos
Anti-H1 intermitente oral (>3 dias por semana)		1 punto
Moderada/severa intermitente	Esteroides intra-nasal	Cromona local	2 puntos
Leve persistente	Esteroides intra-nasal y antiH1	Esteroides intra-nasal- y AntiL	3 puntos
Moderada/severa persistente	Esteroides intra-nasal + antiH1 + antiL	4 puntos

AntiH1: Antihistamina
